# Reduced Expression of the Retinoblastoma Protein Shows That the Related Signaling Pathway Is Essential for Mediating the Antineoplastic Activity of Erufosine

**DOI:** 10.1371/journal.pone.0100950

**Published:** 2014-07-02

**Authors:** Maya M. Zaharieva, Milen Kirilov, Minquang Chai, Stefan M. Berger, Spiro Konstantinov, Martin R. Berger

**Affiliations:** 1 Toxicology and Chemotherapy Unit, German Cancer Research Center, Heidelberg, Germany; 2 Department of Molecular Biology of the Cell I, German Cancer Research Center, Heidelberg, Germany; 3 Department of Molecular Biology, Central Institute of Mental Health, Mannheim, Germany; 4 Laboratory for Molecular Pharmacology and Experimental Chemotherapy, Department for Pharmacology, Pharmacotherapy and Toxicology, Faculty of Pharmacy, Medical University of Sofia, Sofia, Bulgaria; Witten/ Herdecke University, Germany

## Abstract

Erufosine is a new antineoplastic agent of the group of alkylphosphocholines, which interferes with signal transduction and induces apoptosis in various leukemic and tumor cell lines. The present study was designed to examine for the first time the mechanism of resistance to erufosine in malignant cells with permanently reduced expression of the retinoblastoma (Rb) protein. Bearing in mind the high number of malignancies with reduced level of this tumor-suppressor, this investigation was deemed important for using erufosine, alone or in combination, in patients with compromised RB1 gene expression. For this purpose, clones of the leukemic T-cell line SKW-3 were used, which had been engineered to constantly express differently low Rb levels. The alkylphosphocholine induced apoptosis, stimulated the expression of the cyclin dependent kinase inhibitor p27^Kip1^ and inhibited the synthesis of cyclin D3, thereby causing a G_2_ phase cell cycle arrest and death of cells with wild type Rb expression. In contrast, Rb-deficiency impeded the changes induced by eru-fosine in the expression of these proteins and abrogated the induction of G_2_ arrest, which was correlated with reduced antiproliferative and anticlonogenic activities of the compound. In conclusion, analysis of our results showed for the first time that the Rb signaling pathway is essential for mediating the antineoplastic activity of erufosine and its efficacy in patients with malignant diseases may be predicted by determining the Rb status.

## Introduction

The ether lipid analogue erufosine (erucylphospho-N,N,N,-trimethyl-propylammonium, ErPC_3_) is a new antineoplastic agent classified as a third generation alkylphosphocholine (APC) [1]. It exhibits high activity against leukemic cells without affecting the normal hematopoiesis [2]–[5]. It is the first APC that can be administered intravenously because it does not cause hemolysis [6]. Recent studies have shown that erufosine inhibits the activity of protein kinase B (PKB/Akt) and induces apoptosis in a variety of malignant cells [2], [4], [7], [8]. It also targets cell cycle regulators such as the retinoblastoma protein (Rb), p27^Kip1^, transcription factors from the E2F family and cyclin D1 [2], [9]–[13]. The Rb-pathway represents one of the most frequently inactivated signaling axes in human cancers [14]–[18]. The retinoblastoma tumor-suppressor gene *RB1*controls, pending on its phosphorylation status, on one hand the cell cycle progression from G1-to S-phase and on the other hand the activation of apoptosis. Hyper-phosphorylation of the Rb protein releases the S phase-promoting transcription factors from the E2F family and is indicative of the cell’s commitment to proliferate [19], [20]. Accumulation of the cyclin/cyclin dependent kinase (CDK) complexes that follows Rb-inactivation and E2F release is regulated negatively by the INK4 and the Cip/Kip protein families, which block progression through the G1/S phase [21]. The other axis of the Rb-pathway leads to the tyrosine kinase c-Abl, which is an inductor of apoptosis. The pro-apoptotic function of c-Abl has been demonstrated in cultured cell lines, explanted thymocytes and mice embryos [22]–[25]. The activation of c-Abl after DNA damage and p53 activation requires Rb phosphorylation or degradation [22], [26], [27]. Welch and Wang have shown that the C-terminal region of Rb can bind to the Abl tyrosine kinase, leading to the inhibition of its kinase activity [24], [28].

Recently, we have shown that a transient Rb-knockdown impairs the antileukemic cytotoxicity of erufosine in chronic myeloid leukemia cell lines as evidenced by cell viability and clonogenicity assays [29]. Until now, conditions of permanent Rb deficiency have not been investigated as basis of resistance to erufosine, nor were malignant cells originating from other types of cancer used as model to determine the underlying mechanisms of action and resistance to erufosine. The latter is crucially important for the clinical application of erufosine alone or in combination with other drugs in the treatment of malignancies characterized by *RB1* mutation or loss. Therefore, we investigated for the first time to which extent permanent Rb deficiency modulates the cells’ response to erufosine as well as to four classical cytostatic agents used as reference drugs. In addition, we focused on proteins of the Rb signaling pathway, which are involved in cell cycle control, proliferation and induction of apoptosis (p16^Ink4A^, p27^Kip1^, p53, Cdk4, c-Abl, cyclins D and E2), to broaden our knowledge on the mechanism of action of erufosine. Our hypothesis was that Rb deficiency will cause resistance to erufosine by loss of the feedback control between Rb and the related proteins from its signaling pathway. Thus, we generated a stable Rb-knockdown in SKW-3 leukemia T-cells using the lentiviral transduction system pLentilox3.7 (pLL 3.7) and isolated two clones with different levels of reduced Rb-expression that were used as model. Here, we report that the reduced antineoplastic activity of erufosine under conditions of stable Rb-knockdown results from the diminished expression of certain Rb controlled cell cycle regulators, which cause accelerated proliferation and impaired induction of apoptosis in the exposed cell populations.

## Materials and Methods

### Compounds, short-hairpin RNAs and expression constructs

**Figure 1:**
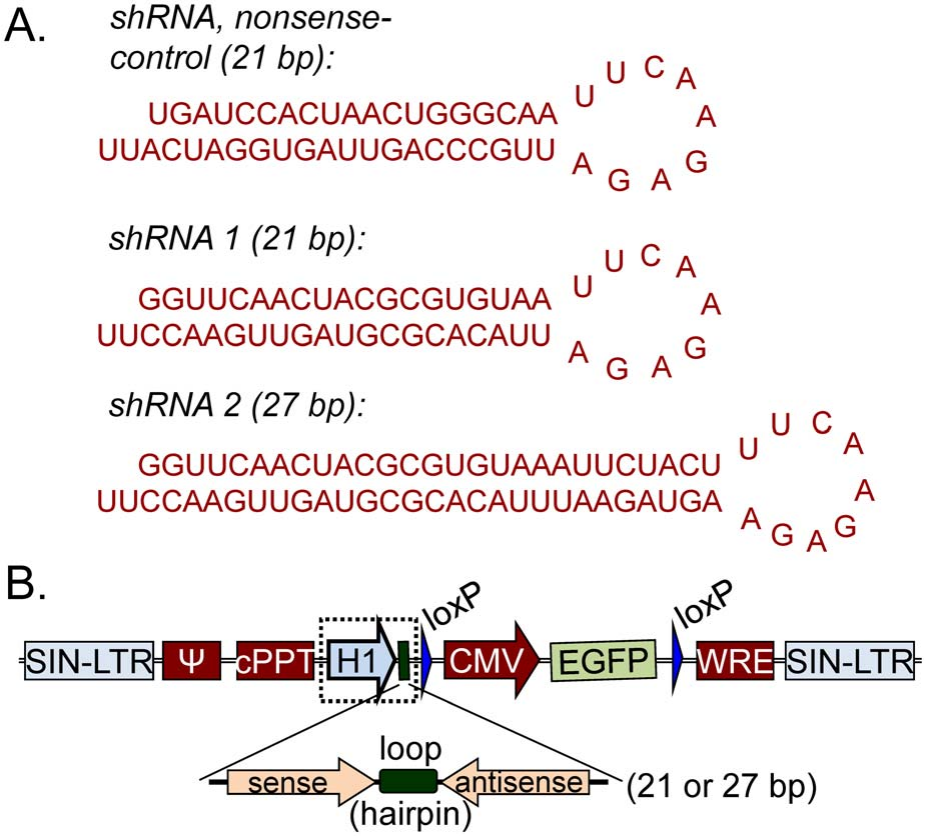
Sequences of the nonsense and antisense shRNA and the pLL 3.7 construct. (A) Sequences of the nonsense and antisense shRNA used for cloning by standard cloning techniques into the lentivirus plasmid pLL 3.7. (B) The shRNA-constructs under the control of the H1-promoter were cloned into the lentivirus plasmid pLL 3.7 after removing the U6-promoter located before the CMV-promoter of the eGFP gene.

Erufosine was kindly provided by Prof. Eibl, MPI-Goettingen, Germany [30] and a solution in 0.9% NaCl was used for all experiments. The cytostatics 5-fluorouracil (Sigma), cytosine arabinoside (Ara C, cytarabine, Sigma), doxorubicin (clinical grade) and cisplatin (Medac) were used as reference drugs. For generating a 21 bp long short hairpin RNA, a target site within the Rb-mRNA was selected *in silico* [31] and the sequence was created with the HiPerformence Design Algorithm of Qiagen (Fig. 1a). Since there were reports, that shRNAs with a longer target site lead to more pronounced knockdown efficiency [32], a 27 nucleotides long shRNA (shRNA Rb-27, Fig. 1a) was constructed in addition [33]. A non-specific21 bp long shRNA was used as a nonsense control (NSO, Fig. 1a). The plasmid pSUPER was kindly provided by Reuven Agami. The self-inactivating vector pLL 3.7 puro-eGFP originated from the laboratory of Luk van Parijs.

### Cell lines and culture conditions

The human T cell leukemia line SKW-3 (ACC 53, DSMZ) and the embryonal kidney cell line HEK-293 (ACC 305, DSMZ) were maintained in RPMI-1640 and Dulbecco’s MEM medium (Invitrogen), respectively, containing 10 to 15% fetal calf serum (Biochrom) and 200 mM L-Glutamine (Invitrogen). Cells were cultured under standard conditions (37°C, humidified atmosphere, 5% CO_2_) and propagated twice weekly.

### Construction of plasmids

DNA fragments, containing the sequence of a particular shRNA (Fig. 1A), were cloned into pSUPER (Fig. S1A) via Bgl II and Hind III strategy (Roche Molecular Biochemicals). Constructs were verified by restriction analysis. The knockdown efficiency of each shRNA was tested on HEK 293-cellsby using Lipofectamine 2000 Reagent (Invitrogen) and normalized to an eGFP expression vector (data not shown). The lentiviral vector pLL 3.7 puro-eGFP was used to enhance the efficiency of the transgenic delivery into the suspension SKW-3 cell line. Briefly, the U6 promoter of pLL 3.7 was replaced by the H1 promoter-shRNA expression cassette of pSUPER via Xba I and XhoI (Roche Molecular Biochemicals) cloning strategy (Fig. S1 and Fig. 1B). Constructs were verified by restriction analysis as well as by sequencing.

### Generation and titer of lentivirus particles

HEK 293-cells were co-transduced (calcium phosphate transfection) with the pLL 3.7 construct containing the respective shRNA expressing cassette, a packaging vector (pMDLg/pRRE) and plasmids expressing the ENV (pMD2.G) and REV genes (pRSVrev). The supernatant was collected, cleared by filtration (0.45-µm pore cellulose acetate filter - Millipore) and concentrated by ultracentrifugation (1.5 h, 25 000 rpm). Pellets were re-suspended in phosphate-buffered saline (PBS, Gibco) and titers were determined by infecting HEK-293 cells with a serial dilution of the virus suspension (∼0.4–5×10^9^ infectious units/ml).

### Lentiviral infection, transduction efficiency and isolation of clones

SKW-3 cells (density: 3×10^5^/ml) were cultured for 24 h, supplemented with 7×10^6^ lentiviral particles/ml (multiplicity of infection, MOI = 3) and 6 µg/ml polybrene, and washed after 24 h with fresh medium. EGFP-positive cells were isolated from each cell population (NSO-, shRNA 1- and shRNA 2 transduced cells) by fluorescence activated cell sorting (FACS) using a FACSVantage DIVA (Becton Dickinson). Thousand cells from each sample were re-suspended in semi-solid medium (0.8% RPMI-methylcellulose and 30% FCS) and plated on Petri dishes (3.5 cm^2^). After 7 days, 24 EGFP-expressing single colonies were isolated from each fraction under a fluorescent microscope.

### Cell cycle analysis

Cell populations were treated with erufosine, washed with PBS and fixed in 70% ethanol-PBS solution for 1 h on ice. Fixed cells were washed twice with ice cold PBS, treated with RNAse A for 30 min at 37°C and stained with propidium iodide 15 min prior to analysis. Cellular DNA content was determined by flow cytometry using the DIVA Program (BD) and ModFit LT software.

### Quantitative RT-PCR (qRT-PCR)

Total RNA was isolated with the RNeasy Mini Kit (Qiagen), reverse-transcribed to cDNA using the SuperScript First-Strand Synthesis System (Invitrogen) and purified with the QIAquick PCR Purification Kit (Qiagen). Levels of Rb-, E2F2-, cyclin D3- and GAPDH-mRNA (primer sequences are given in Table S1) were quantified on a LightCycler480 (Roche) by using LightCycler 480 Probes Master and the human Universal Probe Library (Roche). PCR reactions were performed in 384 well plates (10-µL reaction mixture: 2x LightCycler 480 Probes Master, 100 nM UPL Probe, 200 nM primers, cDNA, deionized water) by thermal cycling conditions: 10 min pre-incubation (95°C), 50 cycles of 10 sec denaturation (95°C) and 30 sec annealing/extension (60°C), followed by 30 sec cooling (40°C). Samples were normalized to the gene glyceraldehyde 3-phosphate dehydrogenase (GAPDH).

### Immunoblot analysis

Samples (2×10^6^ cells) were lysed on ice (100 mMTris-HCl (pH 8.0), 4% sodium dodecyl sulphate, 20% glycerol, 200 mM dithiothreitol, complete protease inhibitor cocktail tablets –Roche), heated to 95°C (10 min) and centrifuged at 8000 *g* (10 min, 4°C). After protein quantification (Pierce Protein Assay, Thermo Fisher Scientific), 30 µg total protein was separated by gradient SDS-PAGE electrophoresis (Invitrogen). Proteins were electro-transferred onto a polyvinylidene-difluoride membrane (Sigma) in a semi-dry Transfer System (GE Healthcare) and identified by specific antibodies [Rb - sc-102, p16^Ink4A^ - sc-81156, p27^Kip1^ - sc-528, p53 - sc-98, Cdk 4 - sc-601, Cyclin D3 - #2936, cyclin E - #4132, c-Abl - #2862, Cleaved Caspase 3 - #9661, Cleaved Caspase 9 - #9501, Cleaved Poly ADP ribose polymerase (PARP)- #9541, and Actin-β - sc-1615] and respective HRP-conjugated secondary antibodies (goat anti-mouse IgG-HRP - sc-2005, donkey anti-goat IgG-HRP - sc-2020, Anti-rabbit IgG, HRP - #7074, Anti-mouse IgG, HRP#7076) according to the manufacturer instructions (Santa Cruz Biotechology, New England Biolabs). Protein bands were detected with an ECL-System (GE Healthcare, Germany). The house-keeping gene *β-actin* was used for normalization of relative protein concentrations as assessed by densitometric analysis (Quantity One, Bio-Rad).

### Cell proliferation and clonogenicity assays

Cell clones were seeded in 96-well plates (3×10^5^ cells/ml) and treated with erufosine (0–32 µM), 5-FU (0–50 µM), cytosine arabinoside (0–2 µM), doxorubicin (0–0.032 µM) or cisplatin (1–16 µM) for 48 h. Cell survival fractions were estimated with the [3-(4,5-dimethylthiazol-2-yl)-2,5-diphenyltetrazolium bromide] (MTT) reduction assay [34], [35]. Briefly, eight samples per concentration were incubated for 3.5 h with MTT (10 mg/ml in PBS) at 37°C. Formazan crystals were dissolved by an equivalent volume of 0.04 N HCl/isopropanol. Absorption was measured by an ELISA reader (Anthos 2001, Anthos Labtec) at 540 nm (reference 690 nm) against a blank solution. For the clonogenicity assay, 7000 cells from each sample were transferred into semi-solid medium 48 h after treatment with 2–16 µM erufosine (0.8% RPMI-methylcellulose, 30% FCS), plated onto 24-well plates (350 µl/well) and incubated 7 days (37°C, 5% CO_2_, humidified air). Colonies (20 or more cells) were scored by an inverted microscope.

### Statistical analysis

All experiments were performed in triplicate. Results from cell viability and clonogenicity assays are presented as percent of the untreated control. IC_50_ values of the drugs were calculated with the GraphPadPrizm program by using nonlinear regression mathematical model “Dose-response inhibition: Log (inhibitor) vs. normalized response, Variable slope” based on the equation Y = 100/[1+10∧((LogIC_50_-X)*HillSlope)]. The significance of differences was analyzed using the Student’s *t*-test (*P<0.05, GraphPadPrizm) and multivariate analysis of variance (M-ANOVA).

## Results and Discussion

Erufosine was shown to induce increased expression, hypo-phosphorylation or fragmentation of the Rb tumor suppressor protein in cancer cell lines [36]. In our previous investigations, a transient Rb-knockdown, which was caused in chronic myeloid leukemia cells, was associated with diminished sensitivity to erufosine, as determined by proliferation and clonogenicity assays [29]. However, the mechanism of this resistance was not defined. The present study aimed to prove that the retinoblastoma protein pathway is central to erufosine’s antineoplastic activity and induction of apoptosis. In a step by step procedure, our hypothesis was investigated in cell clones with different levels of stable Rb-deficiency, which were generated from the T-cell leukemia line SKW-3, chosen because of its select sensitivity to erufosine [36].

### Development of a cell line panel characterized by Rb-deficiency

**Figure 2:**
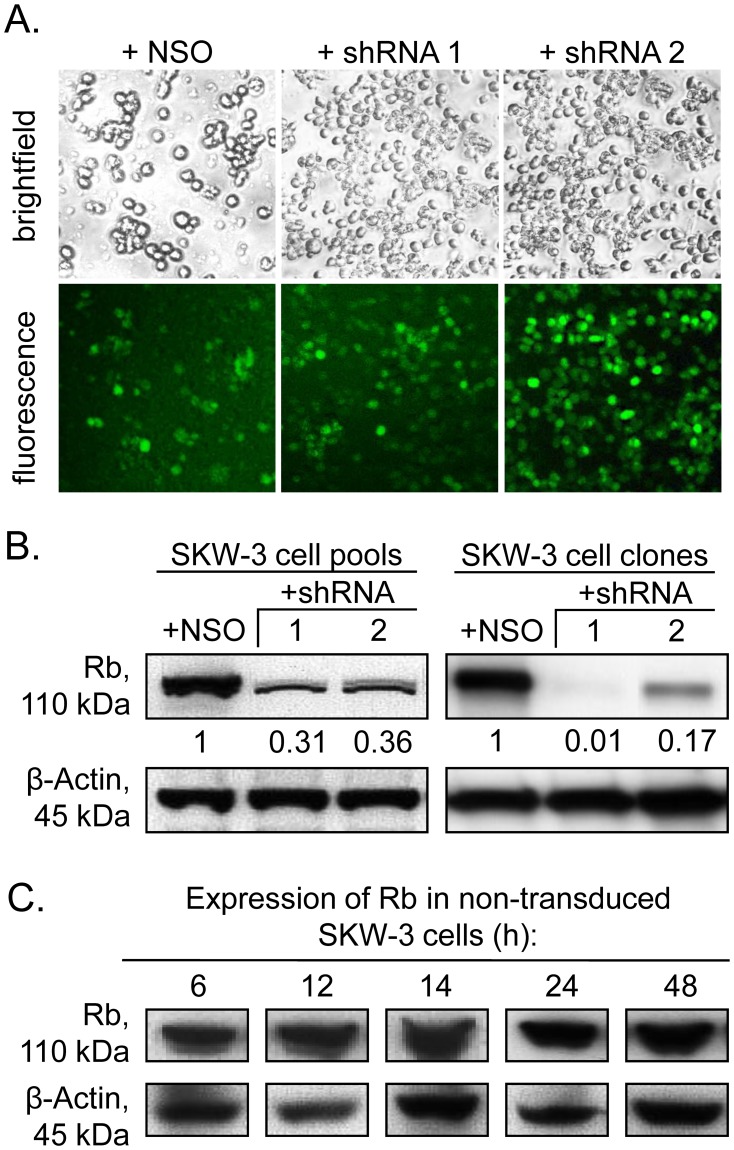
Efficacy of the Rb-knockdown. (A) Fluorescence imaging of SKW-3 cells at 72 h after viral transduction with three different pLL 3.7-constructs (nonsense shRNA – NSO, antisense shRNAs–21 bp, 1 or 27 bp, 2) and Cell Sorting using the eGFP signal. (B) The efficacy of the Rb-knockdown estimated by Western blot before and after selection. The Rb-knockdown on protein level was calculated as percentage of the respective nonsense-control cells after densitometric analysis of the protein bands using the Quantity One 4.6.6 Program (Bio-Rad Laboratories).

The stable Rb-knockdown was accomplished by two shRNA sequences with different length (Fig. 1). The ratios of eGFP expressing SKW-3 cells, isolated by FACS 72 h after viral transduction, were 90% for the NSO control and 99% for each shRNA species (Fig. 2A). The expression of NSO-shRNA did not alter the Rb concentration in transduced as compared to wild type cells by using immunoblot analysis and therefore it was chosen as negative control in all experiments (Fig. 2B and C). In contrast to other reports [37], RNA silencing caused by the longer sequence was not more efficient than that by the shorter one. In fact, the knockdown efficiency of the 27 bp long shRNA 2 was lower than that of the 21 bp long shRNA 1 (69% for shRNA 1 and 64% for shRNA2, Fig. 2B). Clonal selection resulted in isolation of three cell clones (Fig. 2B), i.e. the NSO-shRNA clone exhibiting a wild-type like growth rate (MTT analysis, data not shown), one with 99% Rb knockdown (from shRNA 1) and one with 83% reduction (from shRNA 2) that were used for all subsequent experiments. The two clones, which differed in their residual Rb-levels by 16% (1% versus 17%), allowed to compare Rb dependent changes in cell cycle control and apoptosis after treatment with erufosine between conditions of Rb loss and Rb deficiency.

### Rb-knockdown impairs the antiproliferative effect of erufosine

**Figure 3:**
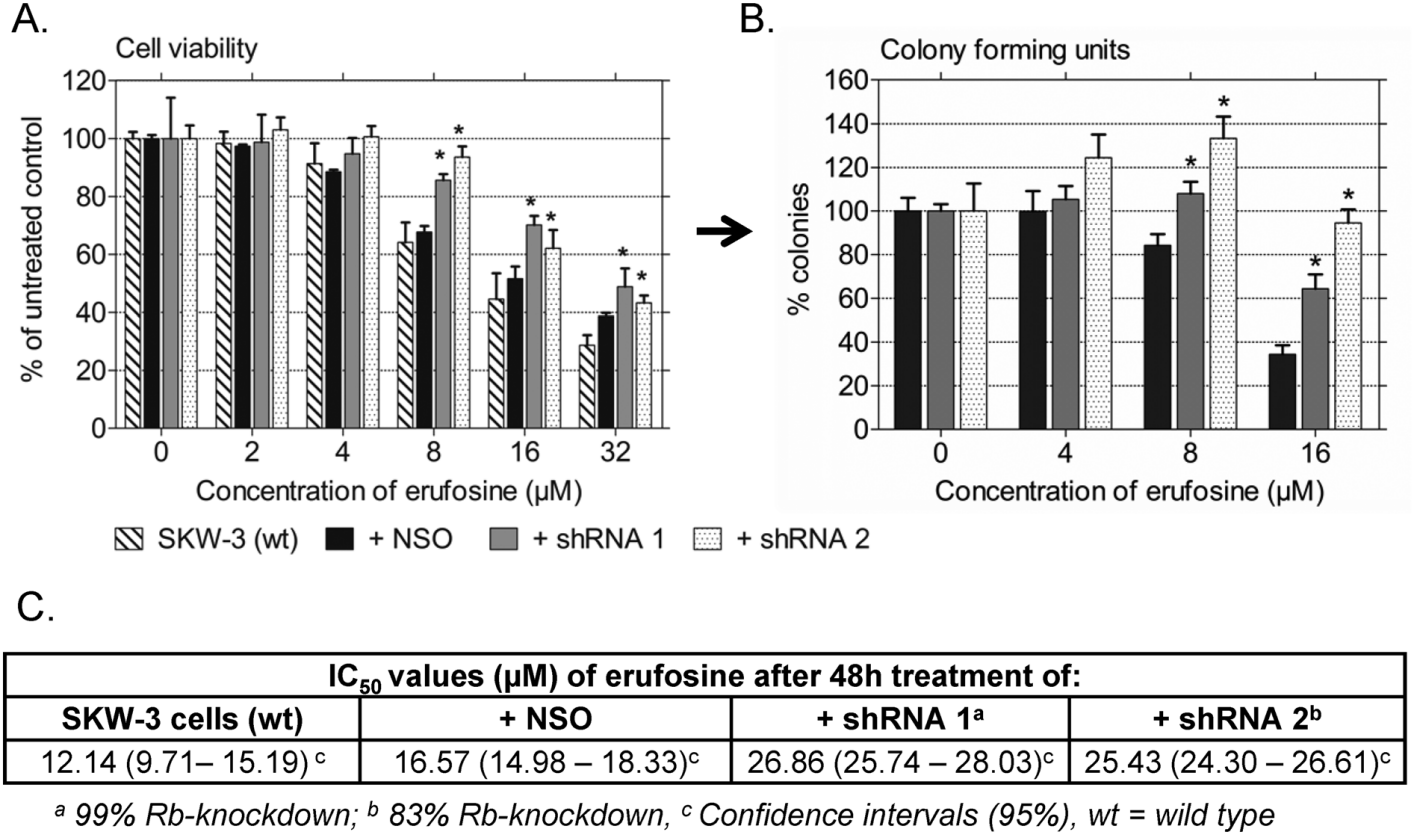
Cytotoxicity and clonogenic activity of erufosine in Rb-deficient cells. The figure depicts the survival (A) or clonogenicity (B) of SKW-3 cell clones with 99% (shRNA1) or 83% (shRNA 2) stable Rb-knockdown after treatment with 2, 4, 8, 16 or 32 µM erufosine for 48 h. A significant difference versus the respective nonsense control is marked by an asterisk (Student’s t-test; p<0.05). Bars denote standard deviation. The table under the graphs gives the IC_50_ values of erufosine after 48 h of treatment as well as the respective 95% confidence intervals.

The strong antileukemic activity of erufosine was described before in studies on malignant cells from patients with different types of leukemia [2], [4], [5], [38]. In our anti-proliferative and anti-clonogenic assays (Fig. 3), erufosine exhibited high cytotoxicity in SKW-3 wild type (IC_50_ = 12.14 µM) and nonsense control cells (IC_50_ = 16.57 µM) without a significant variance between wild type and NSO-transduced cells (Fig. 3, table with IC_50_ values and 95% confidence intervals), whereas loss of Rb or reduced Rb levels caused significantly increased resistance to treatment with erufosine (IC_50_/shRNA 1 = 26.86 µM; IC_50_/shRNA 2 = 25.43 µM), which is in line with the results from our previous study conducted in other types of leukemic cell lines [29]. The surviving cell fraction measured by MTT-assay in both cell populations with Rb-knockdown in response to 8–32 µM erufosine was 15% to 30% higher than in the wild type or NSO control cells resulting in 1.6 fold higher IC_50_ values of erufosine in Rb-deficient cells (Fig. 3A). In addition, CFU-assay was performed after 48 h exposure to erufosine (4–16 µM) to investigate the clonogenic activity of the treated cells with altered Rb expression in comparison to the NSO control. Since there was no significant difference between the IC_50_ values of erufosine calculated after the MTT-assay for the wild type SKW-3 cells and the nonsense transduced control (Table in Fig. 3), the IC_50_ value (16 µM) of the latter was used as control for all subsequent experiments and for treating the cells in the cell cycle and protein expression analyses. Data analysis from the CFU-assay showed that colony formation was stimulated under conditions of Rb-knockdown by 15–70% as compared to the NSO transduced control cells (Fig. 3B), which indicates the well-kept ability of viable but Rb-deficient cells to divide and form colonies in a semisolid medium as well as their accelerated proliferation rate. Both findings give strong evidence that erufosine’s cytotoxic effect markedly depends on the Rb expression status.

**Figure 4:**
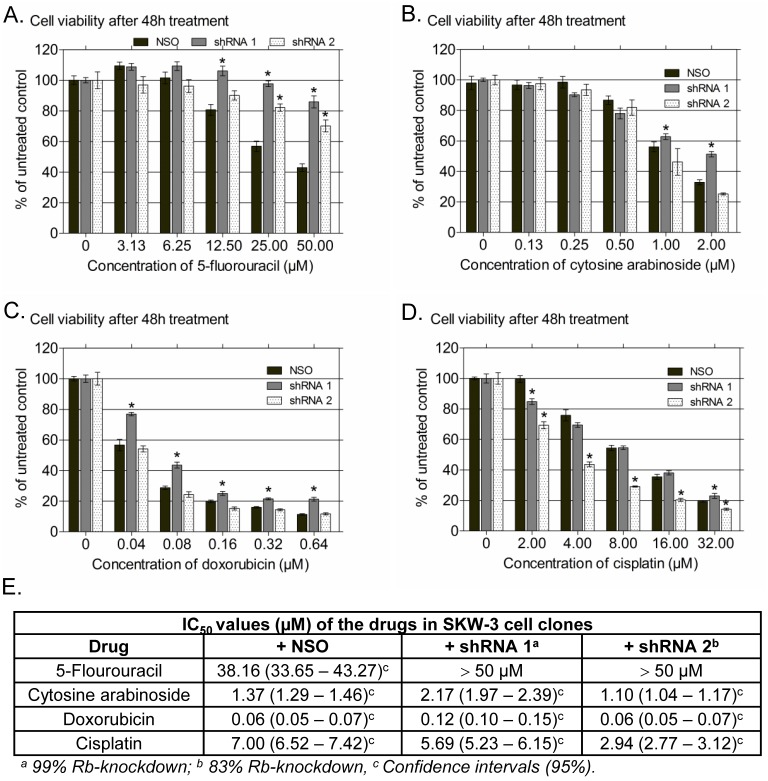
Survival of cells with stable Rb-knockdown after 48 **h treatment with 5-floururacil, cytosine arabinoside, doxorubicin and cisplatin.** SKW-3 cell clones (nonsense control – NSO, cells with 99% Rb-knockdown – shRNA 1 and cells with 83% Rb-knockdown – shRNA 2) were treated with five different concentrations of the four cytostatics. A significant difference versus the respective nonsense control is marked by an asterisk (Student’s t-test; p<0.05). Bars denote standard deviation. The table under the graphs gives the IC_50_ values of the drugs after 48 h of treatment with the respective 95% confidence intervals.

Four clinically approved cytostatics were used as reference drugs for the cell viability analysis, two of which (5-fluorouracil and cytarabine) are pyrimidine analogues belonging to the S-phase specific antineoplastic agents and the other two (doxorubicin and cisplatin) are representatives of the groups of anthracyclines and alkylating agents, respectively, and belong to the phase-nonspecific anticancer drugs [39]. They were chosen because their mode of action is well known from the literature and their activity in Rb-mutant cells has been studied in detail before. Except 5-fluorouracil, the other three drugs showed a less pronounced modulation of their antineoplastic efficacy under conditions of Rb-deficiency as compared to erufosine (Fig. 4, see table under the graphs), which could be explained by their different modes of action. Our results are in line with previous investigations showing that Rb-loss can induce either light to moderate resistance or increased chemosensitivity towards chemotherapy pending on the cytostatic used [15], [22], [40]. In our study, the highest resistance was observed for the anti-metabolite 5-FU, which is an S-phase dependent cytostatic. Its cytotoxic efficacy declined in Rb-knockdown clones by 10%–40% as compared to the nonsense control, irrespective of the Rb-knockdown extent (IC_50_ = 41 µM), which could be explained by the slower growth of the modulated cell populations (data not shown) and by presumably increased levels of thymidilate synthase (TS), the cellular target of 5-FU, as described for cells without Rb [41]. A study performed on adult somatic cells revealed that following spontaneous immortalization and loss of functional p53, 5-FU failed to induce cell cycle arrest despite the presence of Rb [42]. Thus, lower levels of p53 in our Rb-deficient cell clones probably contributed also to the diminished activity of 5-FU. The antineoplastic efficacies of cytosine arabinoside and doxorubicin were slightly decreased by complete Rb-knockdown (shRNA1) only. Ara C also incorporates into DNA, such as 5-FU, and retards chain elongation, but it differs in its mode of action from 5-FU in its ability to inhibit DNA polymerase, both in replication and repair [39]. The resistance to Ara C in cells with complete Rb-knockdown was associated with reduced levels of p53, mutations of which were found to be causal for resistance to Ara C therapy in patients with acute myeloid leukemia (AML) because of impaired apoptosis induction [43]. Doxorubicin, which is a DNA damaging agent, was less effective in cells with full Rb-knockdown but not in the setting with 17% residual Rb-expression. As found by Jackson et al. [44], it induces a senescence-like phenotype in breast cancer cells that involves p53-p21 responses early after drug treatment followed by p130 recruitment to key promoters regulating cell cycle transition. Knockdown of all three Rb family members (Rb, p107 and p130) was required to bypass the downregulation of cell cycle genes because of compensatory roles of p107 and Rb [44]. In line with this study, 83% Rb-knockdown in our experiments was not enough to change the sensitivity of the treated cell population towards doxorubicin. In variance to the other drugs, exposure to cisplatin was associated with increased sensitivity of the clone showing 17% Rb-expression, but no difference of the cell clone with complete Rb-knockdown. Cisplatin is active in all phases of the cell cycle, causes DNA breaks and activates as a consequence the p53 signaling pathway, followed by apoptosis involving the Rb/c-Abl pathway. The activation of p53 depends on the phosphorylation status of Rb, suggesting that higher levels of Rb and c-Abl (as mediator of apoptosis) in cells with 17% Rb expression could be a reason for their increased sensitivity. Reed et al. reported that cisplatin causes significantly decreased cellular viability in Rb-deficient lung cancer cell lines because it deregulates certain Rb/E2F target genes and the G_1_ checkpoint mechanism [41]. Similarly, Seely et al. [45] treated Schwann cells, proficient and deficient in Rb, with cisplatin and observed that a significant fraction of Rb-deficient cells entered the S-phase of the cell cycle at very low concentrations of the drug, in contrast to cells proficient for Rb, which were inhibited in their ability to proliferate. Because this was not observed for fibroblasts examined in their study, they concluded that the sensitivity of cancer cells with Rb-knockdown towards cisplatin depends on the cell type and context. Based on this experiment, the impaired antiproliferative activity of Rb-null and Rb-deficient cells of our cell panel could be due to deregulation of the Rb-dependant G_1_/S entry because of impaired transcription of certain Rb/E2F target genes. To elucidate in detail, how and to what extend the mechanism of erufosine’s antineoplastic activity depends on the Rb status, we performed cell cycle analysis and determined the expression levels of certain Rb-related cell cycle regulators from the Rb signaling pathway.

### Rb-loss inhibits the erufosine-induced G_2_ cell cycle arrest

**Figure 5:**
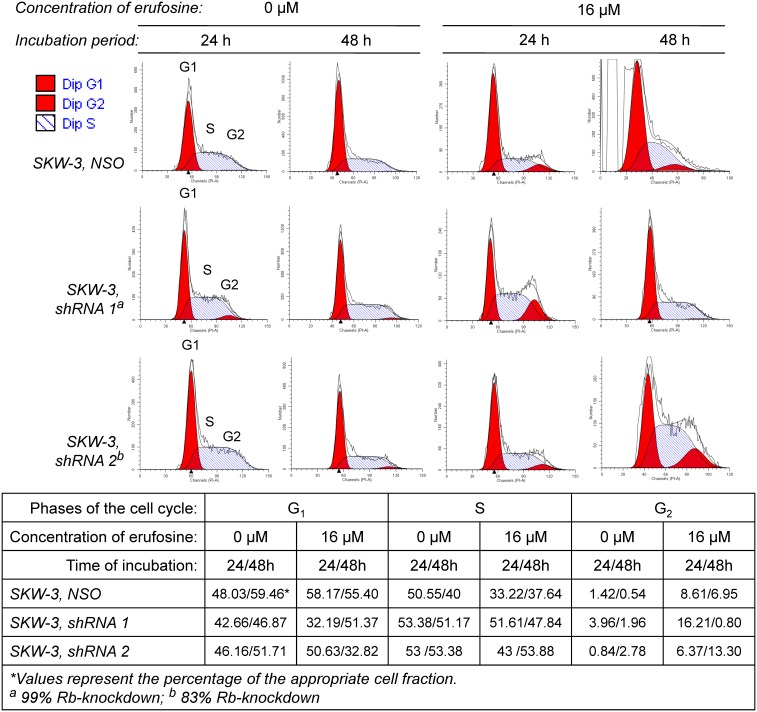
Rb-loss inhibits the erufosine-induced G2 cell cycle arrest. The flow cytometry histograms present the distribution of nonsense- or antisense transduced SKW-3 cell clones with 99% (shRNA 1) and 83% (shRNA 2) Rb-knockdown in G1-, S- and G2-phases of the cell cycle before and after exposure to 16 µM erufosine for 24 and 48 h. The percentage of the cell fractions is calculated with the ModFit LT software and given in the table below the graphs.

Cell cycle analysis was performed to explore the mechanisms underlying the erufosine-mediated cell growth inhibition as well as the resistance of cell clones with Rb-knockdown. Erufosine caused G_2_ arrest in nonsense cells, as was recently found in oral squamous carcinoma cells [8]. Exposure of the NSO control to erufosine led to a 6–14 fold increase in the G_2_ cell fraction at 24 h and 48 h, respectively, which corresponded to a decrease of the S-phase cell fraction from 51% to 33% at 24 h (Fig. 5). Other authors have reported that Rb-deficient cells cannot undergo G1, mid-S or G2 arrest following DNA damage, although they can activate the G2 checkpoint, which is reversible [40]. In line with this finding, cells with Rb-loss (shRNA 1) were characterized in our study by a genuinely enhanced G_2_ cell fraction (4%), which increased 4 fold at 24 h following exposure to erufosine at the expense of G_1_-phase cells but was completely gone at 48 h. Concomitantly, the S-fraction remained unchanged, which could be reason for the enhanced clonogenic activity of Rb-deficient cells and evidence for the well-kept-up ability of the cells to proliferate. Erufosine treatment of cells with 83% Rb-knockdown (shRNA 2) increased the G_2_ fraction at 24 h 8 fold at the expense of the S phase fraction, which dropped from 53% to 43%. The subsequent increase in G_2_ (5 fold) at 48 h after erufosine treatment correlated with a drop in G_1_ phase cells from 50.7% to 32.8%, whereas the S phase cell fraction reached again its initial value (54%). The delayed G_2_ arrest in cells with 83% Rb-knockdown could be due to the higher expression of cyclin D3 (Fig. 5). However, despite the delayed G_2_ arrest, the cell population with 17% Rb-expression continued to proliferate as evidenced by the enhanced colony formation (Fig. 3B). Cell survival and the stunned erufosine-induced G_2_ arrest can be explained by the unchanged S-phase cell fraction due to inhibition of the cell cycle regulators and tumor suppressor proteins p16^Ink4A^ andp27^Kip1^ under conditions of Rb deficiency and the increase in cyclin E2 after treatment of Rb-deficient cells with erufosine (Fig. 5) [14].

### Erufosine antineoplastic activity depends on the Rb signaling pathway

**Figure 6:**
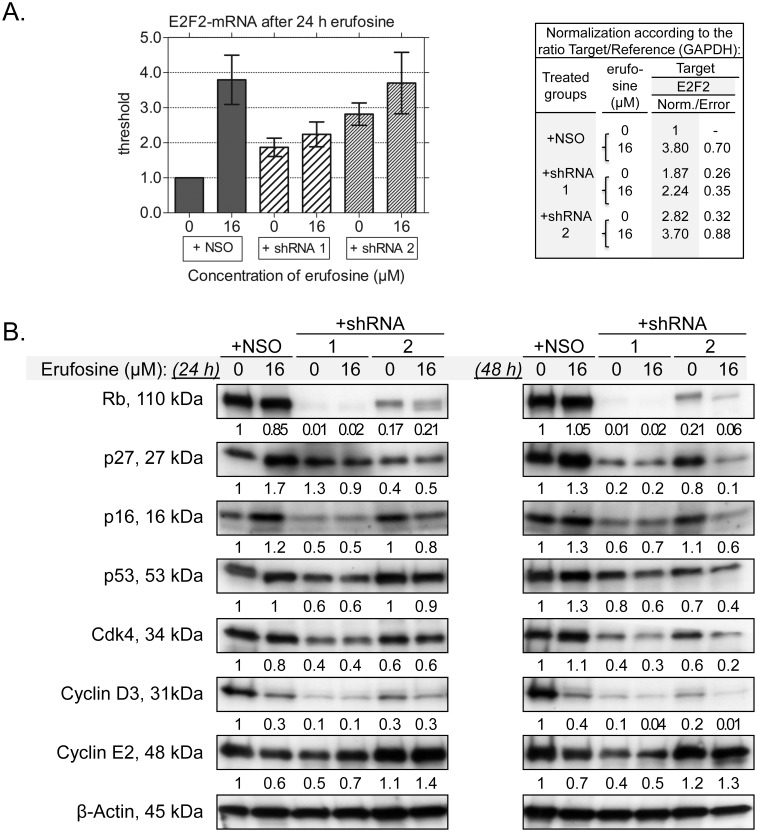
Altered expression levels of cell cycle regulators impair the cytotoxic activity of erufosine by Rb-knockdown. The columns (A) present the expression of the transcription factor E2F2 on mRNA level in SKW-3 cell clones with 99% (shRNA 1) and 83% (shRNA 2) stable Rb-knockdown after treatment with 16 µM erufosine for 24 h. The values and standard errors are calculated after normalization according to the ratio target gene versus reference gene (GAPDH) as shown in the table beside the graphs. The expression of proteins related to the cell cycle regulation before and after treatment with 16 µM erufosine for 48 h is given in panel B. The values under the protein bands denote their intensity compared to the untreated nonsense control and are calculated after densitometric analysis with the Quantity One 4.6.6 Program (Bio-Rad).

The influence of erufosine on Rb-related signal-cascades in nonsense control cells was investigated to determine whether changes in the expression of specific cell cycle regulators are responsible for the erufosine-induced G_2_ arrest and the altered cellular response under conditions of Rb-deficiency. For the first time, we showed that erufosine (16 µM, 24 h) causes a fourfold increase in the expression of the transcription factor E2F2 as assessed by qRT-PCR (Fig. 6A), in concert with a reciprocal down-regulation of cyclin D3 (−70%) and a moderate reduction in cyclin E (−40%) at posttranscriptional level (Fig. 6B). The transcription factor E2F2 has been implicated as a tumor suppressor protein and repressor of T lymphocyte proliferation [46], [47]. It stimulates apoptosis, and its disruption accelerates S phase entry and cell division [48], [49]. Based on this analysis, it could be assumed that the increased expression of E2F2 was one of the reasons for the induction of apoptotic cell death in SKW-3 cells in our study. In some contexts, loss of E2F2 may indicate increased levels of several E2F targets, including cyclin D3, as a result of alterations in the p16-cyclin D-Rb pathway [48], [50]. Cyclins D1, D2 and D3 are important regulators of the G_1_/S entry and their presence is crucial for releasing cells from the G_0_ state [51]. Since no expression of cyclins D1 and D2 was observed in our T-cell leukemia populations (qPCR data not shown), we focused on the expression of cyclin D3. Cyclin D3 was found by other studies to be over-expressed and to cause increased proliferation in acute myeloid leukemia and T- or B-lymphoid leukemia/lymphoma cells [51], [52]. Down-regulation of cyclin D3 was causal for the accumulation of cells in G_2_ and therefore, it is concluded, that the observed cell cycle inhibition caused by erufosine in our experiments is a consequence of the suppression of cyclin D3 (Fig. 6B). In parallel, erufosine increased significantly the expression of the Cdk inhibitor p27^Kip1^ by 70%, whereas levels of Cdk4 remained unchanged. The tumor suppressor p27^Kip1^ contributes to cell cycle arrest [19], [21] and is regulated by ubiquitination and subsequent proteasomal degradation [53], [54]. The levels of p27 appear to be crucial for cell survival and induction of programmed cell death [55]. Its increased levels in response to erufosine treatment suggest that erufosine may act as a proteasome inhibitor and by this way contribute to inhibition of the cell cycle and induction of apoptosis.

In the context of Rb down-regulation, we observed two- to threefold increased mRNA levels of the transcription factor E2F2, which is in line with previous findings that Rb inactivation and E2F over-expression co-exist in tumor cells [46]. The increased levels of E2F2 could be a reason for the apoptotic cell fraction within Rb-knockdown cells (Fig. 6A), as described by others [15], [46]. Cyclin D3 and Cdk4 levels were decreased by 90% and 60%, respectively, while cyclin E2 was moderately reduced by severe Rb-deficiency, but increased in cells with 17% residual Rb-expression (Fig. 6B). Cyclin E2 is over-expressed in tumor-derived cells and accelerates the G_1_ phase [56]. Reduced Rb-levels were associated with diminished amounts of p27^Kip1^, p16^Ink4A^ and p53 in our cell panel, more pronounced in cells with 99% than in those with 83% Rb-knockdown, a status, which is known to cause loss in cell cycle control, deregulation of DNA damage repair and accelerated proliferation [14].

Treatment of Rb-deficient cells with erufosine revealed an altered influence on the studied cell cycle related proteins (Fig. 6B). E2F2 levels remained significantly lower after treatment of cells with severe Rb-knockdown by erufosine, in contrast to the NSO-control and cells with 17% Rb-expression (Fig. 6A). As a repressor of T lymphocyte proliferation, E2F2 is involved in cell proliferation and its decreased expression may contribute to the survival of treated cells with Rb-loss [47]. In contrast to its activity in NSO-cells, erufosine did not enhance in cells with complete Rb knockdown the protein levels of p16^Ink4A^, p27^Kip1^, p53, or Cdk4, nor inhibited the expression of cyclin D3. Low levels of the cell cycle regulators p16 and p27 and the tumor suppressor protein p53 have been correlated with accelerated proliferation and loss of cell cycle control in other studies [14], [40], which explains the resistance of the treated cell population towards erufosine. In cells with 83% Rb-knockdown; however, exposure to erufosine caused a significant suppression of the investigated proteins, which points to their involvement in mediating the cytotoxic effect of the compound. The only exception of this series was the S-phase specific cyclin E2, which showed a slightly increased expression in response to erufosine at 24 and 48 h and may contribute to the increased clonogenicity of the cells after erufosine treatment (Fig. 3). Inhibition of the tumor suppressor proteins p16^Ink4A^ and p27^Kip1^ in combination with increased expression of cyclin E2 is indicative for cell cycle progression and uncontrolled proliferation [56]. These findings correlate with the accelerated clonogenicity of our Rb-deficient cell panel. They are supported by earlier reports that loss or deficiency of Rb expression impairs the chemotherapy response [14], [20], [57], [58].

c-Abl down-regulation by Rb-deficiency is related to impaired erufosine’s activity.

In our study, erufosine (16 µM, 48 h) caused a 30% increase the expression of the tumor suppressor protein p53 in the NSO control, as calculated by densitometric analysis. In Rb-deficient cells, p53 expression was by 30% diminished at 48 h due to the lack of Rb protein and erufosine treatment decreased p53 levels significantly further to 60% and 40% in both cell clones (Fig. 6B). The tumor suppressor protein p53 activates the apoptotic machinery through induction of the release of cytochrome *c* from the mitochondrial intermembrane space resulting in apoptosome formation and activation of caspase 9 [59]. It promotes apoptosis by DNA damage and is associated with Rb-expression levels [14], [40]. Enhanced expression of p53 by DNA damage results in Rb activation and degradation, which is followed by activation of the c-Abl tyrosine kinase, one of the regulators of the apoptotic cell death [22], [26], [27]. The Abl-tyrosine kinase itself can activate the apoptotic function of p53 by phosphorylating Mdm-2 [60]. In addition, it regulates the auto-cleavage of caspase 9 and the activation of various apoptotic factors [61], [62]. Therefore, we assumed that decreased p53 levels would result in an impaired induction of apoptosis in erufosine treated Rb-deficient cell clones. Consequently, the expression levels of c-Abl and the related pro-apoptotic factors caspase 3, caspase 9 and PARP were examined at 48 h of incubation with erufosine to elucidate whether diminished p53 expression is associated with resistance to erufosine. Moreover, the clonogenicity assay was performed after the same incubation period and cells were plated after 48 h in a semisolid medium for further incubation to check their clonogenic ability. In this context, an additional investigation of the apoptotic status of the surviving cell fraction at 48 h was to give improved understanding of the resistance to erufosine.

**Figure 7:**
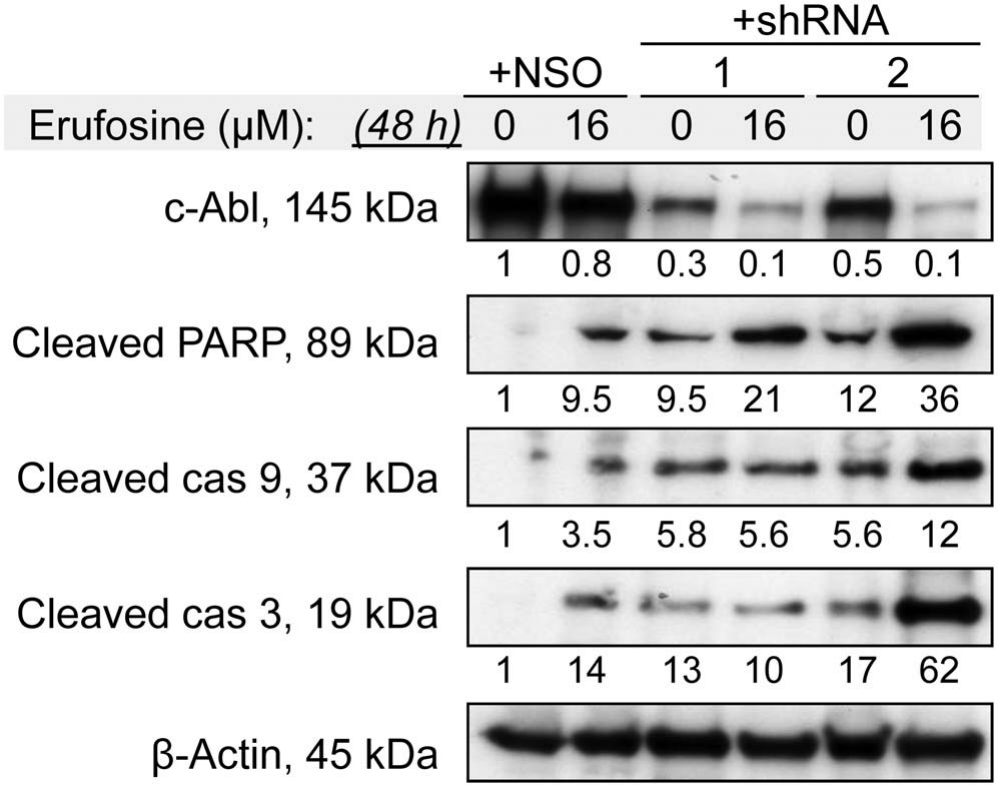
Erufosine-induced apoptosis is inhibited by Rb-loss. The expression of proteins is shown, which are involved in induction of the intrinsic apoptotic pathway, respectively, for nonsense or antisense-transduced cell clones with 99% or 83% Rb-knockdown after exposure to erufosine (16 µM, 48 h). The values under the protein bands denote their intensity compared to the untreated control and are calculated after densitometric analysis with the Quantity One 4.6.6 Program (Bio-Rad).

As shown in Fig. 7, erufosine caused cleavage of caspases 9 and 3, and of PARP in NSO cells, as is indicative of the activation of the intrinsic apoptotic pathway most probably due to the higher expression of E2F2, p27 and p53 (Fig. 6B) [63]. This confirms and extends earlier studies showing that erufosine induces apoptosis in a variety of cell lines [11]–[13]. In the context of Rb-deficiency, reduced levels of c-Abl and activation of various apoptotic factors were observed. The C-terminal domain of the Rb protein binds to the kinase domain of c-Abl and inhibits its pro-apoptotic catalytic activity [22], [24], [26], [28], indicating a negative feedback loop between reduced Rb-levels and c-Abl expression. It has been demonstrated that Rb-knockout embryos exhibited inappropriate cell cycle entry and massive apoptosis in nervous system, lens, liver and muscles [64]. According to Borges et al., in Rb-null mice embryos, the knockout of either one or two *abl* alleles can reduce CNS and liver apoptosis in early embryos suggesting that in Rb^−/−^ genetic background, the pro-apoptotic activity of c-Abl may be enhanced due to the loss of Rb control of the nuclear Abl kinase activity [24]. Other studies have demonstrated that cultured thymocytes derived from *rb^−/−/^abl^−/−^* embryos showed a reduced apoptotic response to TNF-α [23] and to ionizing radiation in *ex vivo* cultures [24]. These facts, in combination with the enhanced expression of E2F2 (Fig. 6A), could be the most probable reason for the presence of a spontaneous apoptotic fraction in the untreated Rb-deficient T-cell clones. Despite the process of spontaneous apoptosis, no enhancement of the apoptotic cell fraction was observed in cells with complete Rb-knockdown after exposure to erufosine (Fig. 7). A plausible explanation for this could be the very low level of c-Abl [24] in combination with the diminished expression of p27, which has been shown to induce apoptosis in haematopoietic cells [55]. This result correlates with the reduced sensitivity to erufosine and increased clonogenic activity of cells with Rb-knockdown in comparison to the NSO control (Fig. 3). A more pronounced pro-apoptotic effect was found in our cells with higher Rb expression resulting in higher levels of c-Abl and p27, thus reinforcing the dependence of erufosine’s activity on the expression of Rb [29], [36]. However, the CFU assay from these cells as well as from cells with complete Rb-knockdown showed high resistance to erufosine. This could be explained by the finding of Borges et al. that the pro-apoptotic function of *abl* exhibits haploid insufficiency, suggesting the requirement of high c-Abl protein levels for the activation of apoptosis [24]. The c-Abl protein was shown to activate apoptosis in an E2F-dependent way through binding of p53 and associating with the Rb-E2F complex [65], thus loss of one *abl* allele may decrease c-Abl protein levels enough to compromise the formation of these pro-apoptotic transcription complexes and the induction of apoptotic cell death [24]. Lower p53 levels in our Rb-deficient cells presumably contributed to inhibition of apoptosis [22], [59], [60] after erufosine treatment, in contrast to Rb-proficient NSO cells with unchanged p53 expression. In accord with these reports, the high resistance of cells with complete or severe Rb-knockdown to erufosine suggests that 17% Rb expression is not sufficient to promote the transcription of enough p27 and c-Abl, which in combination with enhanced levels of cyclin E turned out to be crucial for cell survival.

## Conclusion

The analysis of data obtained in this study clearly indicates that deficiency of the retinoblastoma protein Rb contributes to chemotherapy resistance towards erufosine, due to inhibition of the tumor suppressor factors p16^Ink4A^, p27^Kip1^, p53, of the tyrosine kinase c-Abl, and subsequent enhancement of the S-phase cyclin E resulting in loss of cell cycle and apoptosis control. Finally, the dependence of erufosine’s antileukemic efficacy on Rb expression points to the possibility that its efficacy in patients might be robustly predicted by determining the Rb status.

## Supporting Information

Figure S1 **Cloning strategy of nonsense and antisense shRNA in pSUPER and pLL 3.7.** DNA fragments, containing the sequence of a particular shRNA were cloned into pSUPER via Bgl II and Hind III strategy. The lentiviral vector Pll 3.7 puro-eGFP was used to enhance the efficiency of the transgenic delivery into the suspension SKW-3 cell line. The U6 promoter of pLL 3.7 was replaced by the H1 promoter-shRNA expression cassette of pSUPER via Xba I and XhoI cloning strategy (B, C).
(TIF)

Table S1 **Primers used in the current study (Assay Design Center, Roche) and the number of the respective UPL Probe from the human Universal Probe Library Set (Roche).**
(DOC)
