# Supplementary figures and images for: Reduced Expression of the Retinoblastoma Protein Shows That the Related Signaling Pathway Is Essential for Mediating the Antineoplastic Activity of Erufosine
Source: PLoS One. 2014 Jul 2;9(7):e100950. doi: 10.1371/journal.pone.0100950 (PMC4079453; doi:10.1371/journal.pone.0100950)

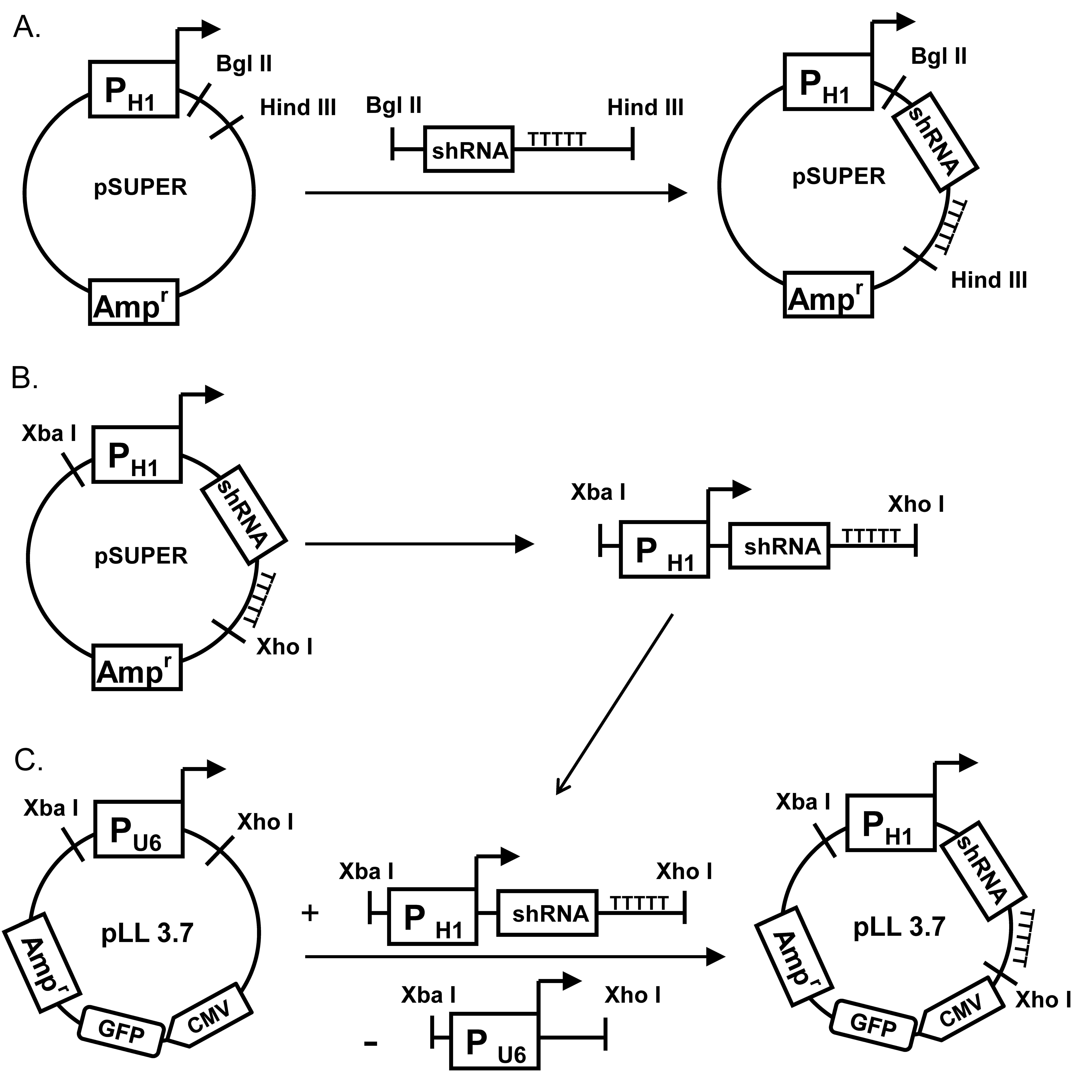

Supplement: Figure S1 — Cloning strategy of nonsense and antisense shRNA in pSUPER and pLL 3.7. DNA fragments, containing the sequence of a particular shRNA were cloned into pSUPER via Bgl II and Hind III strategy. The lentiviral vector Pll 3.7 puro-eGFP was used to enhance the efficiency of the transgenic delivery into the suspension SKW-3 cell line. The U6 promoter of pLL 3.7 was replaced by the H1 promoter-shRNA expression cassette of pSUPER via Xba I and XhoI cloning strategy (B, C). (TIF) [file pone.0100950.s001.tif]
