# Supplementary material for: Reduced Expression of the Retinoblastoma Protein Shows That the Related Signaling Pathway Is Essential for Mediating the Antineoplastic Activity of Erufosine
Source: PLoS One. 2014 Jul 2;9(7):e100950. doi: 10.1371/journal.pone.0100950 (PMC4079453; doi:10.1371/journal.pone.0100950)
Supplement: Table S1 — Primers used in the current study (Assay Design Center, Roche) and the number of the respective UPL Probe from the human Universal Probe Library Set (Roche). (DOC) [file pone.0100950.s002.doc]

**Table S1** Primers used in the current study (Assay Design Center, Roche) and the number of the respective UPL Probe from the human Universal Probe Library Set (Roche).

| **Target mRNA** | **Sequences of the primers** | **UPL Probe** |
| --- | --- | --- |
| Rb | Rb-F (5’-CTT CCT CAT GCT GTT CAG GAG-3’) | #35 |
|  | Rb-R (5’-TGC ATG AAG ACC GAG TTA TAG AAT-3’) |  |
| E2F2 | E2F2-F (5’-GAA GTG CAT CAG AGT GGA TGG-3’) | #23 |
|  | E2F2-R (5’-GAA GTG TCA TAC CGA GTC TTC TCC-3’) |  |
| CD3 | CD3-F (5’-TAC ACC GAC CAC GCT GTCT-3’) | #66 |
|  | CD3-R (5’-GAA GGC CAG GAA ATC ATG TG-3’) |  |
| GAPDH | GAPDH-F (5’-AGC CAC ATC GCT CAG ACAC-3’) | #60 |
|  | GAPDH -R (5’- GCC CAA TAC GAC CAA ATCC-3’) |  |
